
JOURNAL INFORMATION
==============================
NLM Title Abbreviation: Innov Surg Sci
Journal ID: Innov Surg Sci
NLM Journal ID: 101708165
Journal ID: iss

Innovative Surgical Sciences

EISSN: 2364-7485
Publisher: De Gruyter

ARTICLE INFORMATION
==============================
PMCID: PMC11917184
PMID: 40110104
DOI: 10.1515/iss-2025-2009
Article ID: iss-2025-2009
Article version: 1
Subjects: Article

Abstracts DGT

Electronic publication date: 2025 Mar 19
Publication date: 2025 Mar
Volume: 10
Issue: Suppl1
First page: 382
Last page: 387
Copyright: © 2025 by Walter de Gruyter Berlin/Boston
Copyright year: 2025
Copyright holder: De Gruyter
License: This work is licensed under the Creative Commons Attribution 4.0 International License.
License URL: https://creativecommons.org/licenses/by/4.0/

Page count: 6

==============================

Abstracts – DCK 2025 – 142nd Congress of the German Society of Surgery – Munich, March 26–28 • DOI 10.1515/iss-2025-2009
Innov Surg Sci 2025; 10 (Special Suppl 1): s382–s387

**Tracheal membrane laceration after tracheostomy: progressive nightmare under long-term ventilation. Complex repair with vvECMO support.**
ID: 97

**Kategorie: DGT - ECMO in der Thoraxchirurgie**

Stefan Welter[1], Varun Gupta[1], Dietrich Stockhausen[2], Dany Balke[1], Wojciech Dudek[1]

[1]*Hemer, Lungenklinik Hemer, Thoraxchirurgie, Hemer, Deutschland*, [2]*Soest, Marienkrankenhaus Soest, Thoraxchirurgie, Soest, Deutschland*

**Background:**
Iatrogenic tracheal membrane laceration (TML) has been widely described in terms of its acute management, particularly as a complication after oral intubation. Iatrogenic TML during percutaneous dilatational tracheostomy - or in rare cases during surgical tracheostomy - is a problem that has gone largely unnoticed to date. We present a case, that offers a combination of almost all thinkable complications of a posterior tracheal wall injury. Its long-term development turned out to be a permanently life threatening condition.

**Materials and methods:**
Recurrent asphyxia occurred in a 43-year-old women under long-term ventilation due to irreversible hypoxaemic respiratory insufficiency. ICU admission in our hospital was required because of repeated events of sudden blockade of the ventilation chain with hypoxic and hypercapnic coma due to mediastinal dislocation of the tube tip. The patient herself inflated the tube cuff with up to 120 mmHg intending to stop air leakage and prevent another dislocation.

Status on admission: The tracheotomy entrance was extensively dilated longitudinally and transverse. The surrounding skin was severely inflamed and partially ulcerated due to permanent leakage of purulent bronchial secretions. The patient reacted with severe panic attacks whenever there was an alarm on the ventilator due to airway obstruction. The cuff was permanently blocked with pressures above 80 mmHg. For fear of yet another acute blockage of the cannula during movements, the patient strictly refused any mobilization and demanded sedative medication at short intervals. She remained in a strictly supine position.

A flexible bronchoscopy demonstrated a 6 cm O-shaped tracheal posterior wall lesion covered with granulation tissue. At the distal end there was a granulated false way into the posterior mediastinum.

**Results:**
Sufficient oxygenation could be established with a vv-ECMO with cannulation of the right jugular vein and the right femoral vein and a blood flow of 4.5l/min. The access of the trachea was achieved with a collared incision inverted T-shaped to excise the infected skin margins. After exposure and mobilization of the straight neck muscles, the cricoid was found to be destroyed and pushed inwards in a U-shape. The first cartilage ring consisted of soft granulation tissue only. Distally, the trachea was firmly adhered to the connective tissue around the brachiocephalic artery and vein. The posterior wall consisted of granulation tissue, and the spinal column could be touched directly underneath. The trachea was then transected at the lower edge of the tracheostomy opening. The posterior wall of the trachea was sharply dissected, exposing the esophagus, the spine and the lateral edges of the posterior tracheal wall. The granulation tissue was removed from the anterior longitudinal ligament using a sharp spoon. The posterior membrane was then sutured and adapted continuously with resorbable monofilament polyglycolic acid suture 3/0 (Maxon™, Covidien, Medtronic, USA). A continuous ¾ anastomosis of the trachea was then performed in the posterior wall area with 4/0 Maxon™ and the side walls with 3/0 Maxon™ single stitches. The effect was a signifficantly narrowed opening of the tracheostomy entrance. With low pressure in the cuff, the trachea was already airtight. No air leakage, no blockage of the cannula, and wound healing with minimal secretion next to the cannula was recorded over the following 4 months.

After one week, the tracheoflex cannula could be replaced by an 8.0 mm rigid speaking cannula with subglottic suction (Tracoe twist plus, TRACOE medical GmbH, Germany).

Open Access. © 2025 The Author(s), published by De Gruyter. This work is licensed under the Creative Commons Attribution 4.0 International License.

**Conclusion:**
Iatrogenic TML due to tracheostomy may be unnoticed during the procedure. This case illustrates the possible long term consequences in a downward spiral. Only a complex reconstruction of the trachea could eliminate the lethal risk that the patient was permanently in. In this case, vvECMO support was the ideal method to gain a free surgical field, to provide a safe oxygenation in severe pulmonary insufficiency and to help carry out this complex intervention on central airways.

Early identification and repair of TML during tracheostomy may prevent a vicious circle of recurrent dislocation of the tracheostomy cannula with a permanent lethal risk.

**Transmural tracheal lipoma**
ID: 583

**Kategorie: DGT - ECMO in der Thoraxchirurgie**

Bennet Steudel[1], Katrin Hornemann[1]

[1]*Kerckhoff-Klinik, Bad Nauheim, Deutschland*

**Background:**
Tracheal tumors are rare diseases. Of all malignant thoracic tumors only 0.1-0.4% are located in the trachea. For tracheal lipomas the incidence is only 0.1% of all lung tumors. Characteristically diagnosis is confirmed in an advanced state, when they already cause central airway obstruction.

Due to the low numbers of such tumors there are no standardized treatment concepts.

**Materials and methods:**
We describe a 77-year-old man with a tumor on the lateral wall of the distal trachea, shortly before the carina in direction to the right main bronchus. First detection of the tumor in a cardiac ct-scan. A bronchoscopy was performed with biopsy of lymph node in position 10 by EBUS. Histopathology showed no sign for malignancy. The tumor was classified, based on the tumor presentation in the bronchoscopy, as being most likely a carcinoid. Due to the high vascularisation of the tumor and possible bleeding no biopsy was performed. Further diagnostics showed no sign for cranial metastasis and no suspicious FDG enhancement.

The tumor was resected via right-sided thoracotomy and under the support of a veno-venous extracorporeal membrane oxygenation system (ECMO). The ECMO was successfully weaned in the OR and the patient transferred to the ICU after extubation.

**Results:**
Histopathology showed a lipoma with extramural and transmural growth (image 1). The tumor was resected in total (image 2). The tracheal defect could be repaired by a primary suture protected by a pedunculated pericardial patch.

**Conclusion:**
Lipomas occur very rarely in the respiratory tract. In the literature an incidence is described with only 0.1% of all lung tumors. Patients often present by clinical symptoms like dyspnoea, cough or wheezing.

In general these tumors are treated by a surgical or endoscopic resection.

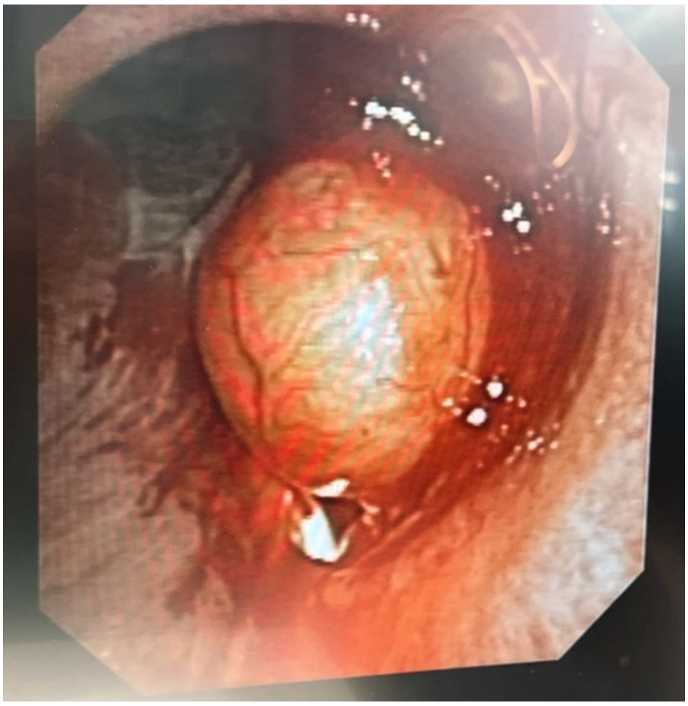

Trachea

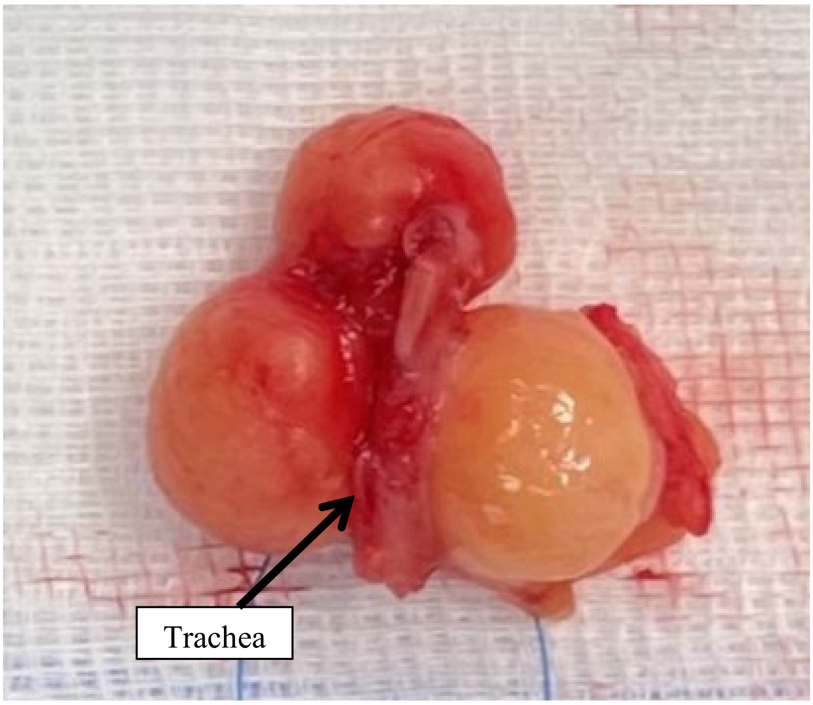

### Surgical Outcomes and Pathological Response after Neoadjuvant Immunochemotherapy followed by Lung Resection in Patients with Non-Small Cell Lung Cancer: A Real-World Evaluation

ID: 386

**Kategorie: DGT - Lungenresektion nach neoadjuvanter Chemo-Immuntherapie**

Tuan Huynh[1], Ansgar Dellmann[2], Tielko Seeba[3], Thomas Bitter[3], Hendrik Eggers[4], Jürgen Krauter[4], Mindia Gulua[1], Wolfgang Harringer[1], Brooke Thorton[1], Ruoyu Zhang[1]

[1]*HTG-Chirurgie, Braunschweig, Deutschland*, [2]*Pathologie, Braunschweig, Deutschland*, [3]*Pneumologie, Braunschweig, Deutschland*, [4]*Hämatologie & Onkologie, Braunschweig, Deutschland*

**Background:**
CheckMate-816 and KEYNOTE-671 trials have demonstrated the significant survival advantage of neoadjuvant immunochemotherapy compared to chemotherapy alone in patients with advanced NSCLC. We sought to evaluate the efficacy of the novel therapy regimen in terms of surgical outcomes and pathological response using real-world data.

**Materials and methods:**
Between February 2023 to July 2024, 15 patients diagnosed with resectable advanced NSCLC at our institution received neoadjuvant immuno-chemotherapy in accordance with either CheckMate-816 (platinum-based chemotherapy plus nivolumab, n=13) or KEYNOTE-671 trials (platinum-based chemotherapy plus pembrolizumab, n=2). Of the following patients, 14 underwent anatomic lung resection, and one was excluded due to tumor progression, making them ineligible for further surgical treatment. The postoperative outcomes and pathological response were reviewed.

**Results:**
Demographics of the 14 patients included in the final analysis consisted of 7 men and 7 women, with a mean age of 66. There were 6 patients with diagnosed adenocarcinoma (42.9%) and 6 patients with squamous cell carcinoma (42.9%). Clinical disease staging varied amongst the included patients and consisted of one patient with stage IIB (7.1%), 6 patients with IIIA (42.9%), 6 patients with IIIB (42.9%), and one patient with stage IVA (7.1%). Surgical procedures included 3 standard lobectomies (21.4%), 10 extended lobectomies (71.4%) and one pneumonectomy (7.1%). There were no postoperative mortalities. Postoperatively, cardiopulmonary complications occurred in 2 (14.3%), including pneumonia and atrial fibrillation in both patients. Complete resection (R0) was achieved in all patients. Complete and major pathological response were found in 5 (35.7%) and 3 (21.4%) patients, respectively.

**Conclusion:**
Surgical resection following neoadjuvant immunochemotherapy is feasible and safe. Our real-world evidence demonstrated a higher rate of complete pathological response than that published in the CheckMate-816 and KEYNOTE-671 trials (24% and 18.1% respectively). The results encourage the recommendation for neoadjuvant immunochemotherapy as a standard regimen for resectable advanced NSCLC.

**Perioperative Predictive Factors for Tumor Regression and Survival in Non-Small Cell Lung Cancer Patients Undergoing Neoadjuvant Treatment and Lung Resection**
ID: 700

**Kategorie: DGT - Lungenresektion nach neoadjuvanter Chemo-Immuntherapie**

Fuad Damirov[1], Mircea Gabriel Stoleriu[2], Gerhard Preissler[1]

[1]*Robert-Bosch-Krankenhaus, Schillerhöhe Lungenklinik, Abteilung für Thoraxchirurgie, Stuttgart, Deutschland*, [2]*Asklepios Lungenklinik Gauting, Abteilung für Thoraxchirurgie, Gauting, Deutschland*

**Background:**
The aim of our study was to identify predictors for the effectiveness of tumor regression in lung cancer patients undergoing neoadjuvant treatment and major lung cancer resections in order to improve the perioperative risk stratification and patient management.

**Materials and methods:**
Patients admitted between 2016 and 2022 at the Department of Thoracic Surgery for neoadjuvant treatment and lung cancer resections were included into the study. Based on the histology of the primary tumor, patients were categorized into two groups: lung adenocarcinoma group (LUAD), and squamous cell carcinoma group (SQCA). The relationship between intraoperative tumor regression score (I: >95% vital tumor cells, IIa: >10% vital tumor cells, IIb: <10% vital tumor cells, III-no vital tumor cells) upon neoadjuvant treatment and well-established perioperative parameters (laboratory, radiological, histological and clinical comorbidities) was analyzed by logistic regression models and Kaplan Meier statistics.

**Results:**
Ninty-five patients with non-small cell lung cancer (33.7 % female, median age 64.20 [57.90; 69.06] years, 44.2% older than 65 years, BMI 24.54 [22.16; 27.04] kg/m$^2$, PY 35.00 [10.00; 50.00]) were included in the study. Fifty-eight (61.1%) and 37 (38.9%) patients were included in the LUAD and SQCA groups, respectively. Nine (9.5%), 56 (58.9%) and 30 (31.6%) patients were categorized with a tumor regression score of I, II and III after neoadjuvant treatment, respectively. In multivariable analyses, histology of the primary tumor (SQCA), lymph node size in the preoperative PET-CT scan (>1.7cm) and tumor size (Delta before-after neoadjuvant treatment >2.6 cm) independently predict effectiveness of tumor regression (OR, [95% Confidence interval, p-value] of 6.88 [2.40-19.77, p<0.0001], 3.13 [1.11-8.83, p=0.0310]; 3.76 [1.20-11.81, p=0.0233], respectively). The tumor size upon neoadjuvant treatment (delta >2.6 cm) was identified as independent predictor of improved Overall survival (84.8% vs 44.0%, p=0.0270, mean 5y-OS 49.8%).

**Conclusion:**
In patients undergoing neoadjuvant treatment and major lung cancer resections, histology of the primary tumor, lymph node and tumor size upon neoadjuvant treatment predict effectiveness of tumor regression. Assessment of tumor size before and after neoadjuvant treatment might help to identify high risk patients with decreased survival and to improve the patient management and care.
